# Electrospun Coaxial Fibers to Optimize the Release of Poorly Water-Soluble Drug

**DOI:** 10.3390/polym14030469

**Published:** 2022-01-24

**Authors:** Yubo Liu, Xiaohong Chen, Yuyang Liu, Yuhang Gao, Ping Liu

**Affiliations:** 1 School of Materials and Chemistry, University of Shanghai for Science & Technology, Shanghai 200093, China; 191370146@st.usst.edu.cn (Y.L.); cxh992@usst.edu.cn (X.C.); 192432630@st.usst.edu.cn (Y.L.); 193742737@st.usst.edu.cn (Y.G.); 2 Shanghai Engineering Technology Research Center for High-Performance Medical Device Materials, Shanghai 200093, China

**Keywords:** coaxial electrospinning, fibers, drug release, poorly water-soluble drugs, polyvinylpyrrolidone

## Abstract

In a drug delivery system, the physicochemical properties of the polymeric matrix have a positive impact on the bioavailability of poorly water-soluble drugs. In this work, monolithic F1 fibers and coaxial F2 fibers were successfully prepared using polyvinylpyrrolidone as the main polymer matrix for drug loading and the poorly water-soluble curcumin (Cur) as a model drug. The hydrophobic poly (3-hydroxybutyric acid-co-3-hydroxyvaleric acid) (PHBV) was designed as a blank layer to change the hydrophilicity of the fiber and restrain the drug dissolution rate. The curved linear morphology without beads of F1 fibers and the straight linear morphology with few spindles of F2 fibers were characterized using field-emission environmental scanning electron microscopy. The amorphous forms of the drug and its good compatibility with polymeric matrix were verified by X-ray diffraction and attenuated total reflectance Fourier transformed infrared spectroscopy. Surface wettability and drug dissolution data showed that the weaker hydrophilicity F2 fibers (31.42° ± 3.07°) had 24 h for Cur dissolution, which was much longer than the better hydrophilic F1 fibers (15.31° ± 2.79°) that dissolved the drug in 4 h.

## 1. Introduction

In recent years, drug delivery systems have attracted widespread attention due to their ability to deliver the correct number of drugs to the proper place [1,2,3,4,5], especially for poorly soluble drugs. More than two-thirds of promising drugs on the market have limited biomedical functions due to dissolution problems [6,7,8]. Thus, many drug delivery systems are exploited to improve the bioavailability of poorly soluble drugs, such as nanoparticles [9,10,11,12,13], hydrogels [14,15], 3D printing [16,17], electrospun fibers [18,19,20,21], nanocapsules [22], and microspheres [23]. Among many drug delivery systems, electrospinning, a “one-step” process with cheap raw materials, shows remarkable potential in the delivery of poorly soluble drugs in recent studies.

Electrospinning is a common method to fabricate drug-loading fibers [24]. Working fluids are gradually stretched into elongated solid fibers with the help of an electrostatic field force [25,26,27]. The diameter of fibers generally ranges from tens of nanometers to several micrometers due to the influence of polymers, solvents, and other external conditions [28]. During this process, drugs are fixed on homogeneous fibers without destroying its own active molecular structure. Compared with other drug delivery systems, electrospun fibers have several advantages. The high specific surface area of fibers is beneficial for drug dissolution. The required drug dissolution process can be customized in accordance with the composition and structure of the fibers, thereby manipulating the controlled dissolution of the drug [7,29,30,31].

More than 200 kinds of polymers have been explored as electrospun fibers until now [32,33,34]. However, drugs are usually selective to solvents and polymers, which remarkably limits the choice of polymers. In accordance with the hydrophilic properties of the drugs, the polymer matrix can be divided into hydrophilic polymers and hydrophobic polymers. Given that the main solvent is deionized water, hydrophilic polyvinyl alcohol (PVA), polyethylene oxide (PEO), and gelatin are suitable for loading water-soluble drugs [35,36,37]. Fortunately, polyvinylpyrrolidone (PVP) is a special hydrophilic polymer for loading most drugs (hydrophilic or hydrophobic). PVP can quickly release the painkiller ibuprofen and promote the oral efficacy of poorly water-soluble drugs [38]. The most common drug-loading hydrophobic polymers are ethyl cellulose ether (EC), cellulose acetate (CA), polycaprolactone (PCL), and poly (lactic-co-glycolic acid) (PLGA), which lead to long-term drug dissolution performance [39,40,41]. In addition, poly (3-hydroxybutyric acid-co-3-hydroxyvaleric acid) (PHBV) is a normal component of human blood, as biomedical materials produced by bacteria using starch as a raw material. PHBV is gradually explored as a new type of drug delivery carrier to release drugs in recent years [42]. Keskin’s group proposed a strategy to control the release of hydro-phobic antibiotics using PCL/PHBV fibers. Fibers have achieved a drug dissolution time of 21 h, but only 22% of drugs were released (due to hydrophobic drugs) [43]. Yu’s group added a functionalized cellulose nanocrystalline to PHBV fibers, which enhance the crystallinity and hydrophilicity of PHBV and achieved the continuous dissolution of tetracycline hydrochloride [42].

The most common preparation method of drug-loading fibers is to blend polymer and drug active molecules. Ranjbar-Mohammadi’s group described the electrospun Cur-loaded PCL/gum tragacanth (GT) fiber products and exhibited a 20-day sustained release [44]. Perumal’s group developed the Cur-loaded blend nanofibers of poly (lactic acid) (PLA) and hyperbranched polyglycerol (HPG) with a high hydrophilicity and swelling properties, and the fibers exhibited a 72 h drug dissolution behavior [45]. However, such single-fluid electrospinning encapsulated drug-loading products inevitably show burst release, thus causing side effects [46]. Coaxial electrospinning is a suitable process to fabricate unique core–shell fibers combined with hydrophilic or hydrophobic polymers (different physicochemical properties) to control the release of drug active molecules [47,48]. Zhao’s group fabricated the metronidazole-loaded PCL/Zein core–shell fibers by coaxial electrospinning, and the drug dissolution time is prolonged from 18 h to more than 72 h [49]. Mo’s group encapsulated the biologically active essential oil as a sheath inside the PCL layer and provided a drug dissolution profile for 3 days [50].

In this work, drug-loaded monolithic and core–shell fibers are successfully fabricated by electrospinning to achieve the controlled release of poorly water-soluble drugs by using hydrophilic PVP and hydrophobic PHBV. Curcumin (Cur), a poorly water-soluble model drug, is a Chinese medicinal material that is recognized as beneficial to the human body [51,52]. Different hydrophilic polymer matrices can have a positive effect on the release of poorly soluble drugs, thereby improving the biocompatibility of drugs. PVP is a recognized polymeric matrix for the loading and release of poorly water-soluble drugs in a short time. Considering the side effects caused by the massive accumulation of Cur, PHBV is designed as a protective layer to delay drug release. Moreover, the characterization and description of fabricated drug-loading fibers in this study and the respective release mechanism are discussed in detail.

## 2. Materials and Methods

### 2.1. Materials

PVP K90 (white powder, Mn = 130,000) and poly (3-hydroxybutyric acid-co-3-hydroxyvaleric acid) (PHBV, natural origin, PHV content = 8 mol%) were obtained from Sigma-Aldrich Trading Co., Ltd. (Shanghai, China). 1,1,1,3,3,3-Hexafluoro-2-propanol (HFIP, C_3_H_2_F_6_O, 99%), ethanol, curcumin, Tween 80, and phosphate-buffered saline (PBS) buffer were purchased from Sinopharm Chemical Reagent Co., Ltd. (Shanghai, China). All the above chemical reagents were not further purified before preparing the spinning solution.

### 2.2. Electrospinning

**Figure 1:**
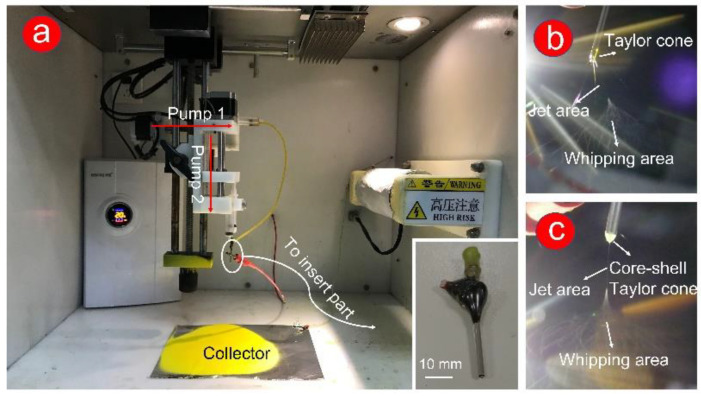
Digital photos of the coaxial electrospinning process. (**a**) Instrument and environment for the preparation of coaxial electrospun F2 fibers, including humidity and temperature control system, pumps, silicone tube, collector, and spinneret. The insert picture shows an enlarged clear picture of homemade spinneret; (**b**) the process of fabricating F1 fibers; (**c**) the process of fabricating F2 fibers.

In this work, two solutions were prepared for electrospinning. One was 0.7 g PVP and 0.2 g Cur in 10 mL ethanol, and the other was 0.9 g PHBV in 10 mL HFIP. The two solutions were stirred for 2 h in a water bath maintained at 50 °C to ensure full dissolution. Solutions were loaded in a 10 mL syringe with a 19 G needle or a homemade spinneret, and a constant voltage was provided by an integrated spinning machine (YFSP-T, Tianjin Yunfan Technology Co., Ltd., Tianjin, China). As shown in Figure 1, the spinning device was transformed into a vertical collection, and the distance between needle (or spinneret) and collector was 13 cm. The internal temperature and humidity were stabilized at 20 °C and 45% by the dehumidification temperature control system.

Two drug-loaded fibers (i.e., F1 and F2) were fabricated in this work. F1 was prepared using 7% (*w*/*v*) PVP with 2% (*w*/*v*) Cur at 0.9 mL·h^−1^, and 7 kV. F2 was prepared by coaxial electrospinning and the homemade spinneret. The core layer was PVP and Cur with a flow rate of 0.9 mL·h^−1^, and the shell layer was PHBV with a flow rate of 1.4 mL·h^−1^ (Page S-2 in Supplementary Materials).

### 2.3. Characterization

#### 2.3.1. Surface Morphology of Fabricating Fibers

The morphology of the prepared fibers was observed using field-emission environmental scanning electron microscopy (FESEM, Quanta FEG 450, Hillsboro, OR, USA) at a working distance of 13.4 mm and an applied voltage of 30 kV. Prior to observation, fibers have been coated with a layer of gold in a nitrogen atmosphere to obtain conductive properties by using the sputtering coating instrument (Q150T ES, Nanjing, China). The average diameter of 100 fibers in SEM images was estimated using the NIH Image J software (version 1.52a, Bethesda, MD, USA).

#### 2.3.2. Phase and Compatibility Analyses

The phase analysis of the fibers was based on the intensity and position of the peak in the X-ray diffraction (XRD) pattern. Fibers were flattened by the cover glass on the sample stage, and the XRD patterns were obtained using a Bruker X-ray diffractometer (Bruker—AXS, Karlsruhe, Germany) at 5° per min from 10° to 70°. Fibers were subjected to attenuated total reflectance Fourier-transformed infrared spectroscopy (PerkinElmer Fourier transform infrared spectroscopy; Billerica, MA, USA). The IR spectra were scanned eight times at a speed of 2 cm^−1^ and range of 500–4500 cm^−1^.

#### 2.3.3. Interface Wettability Test

Fiber mats were cut into 20 mm × 60 mm rectangles and glued on the glass slide smoothly. The JC2000C1 interface tension measuring instrument (Shanghai, China) was used to conduct the water contact angle tests of the prepared samples. At least six droplets of each sample were used, and the average water contact angle was calculated using the ImageJ software (Bethesda, MD, USA).

#### 2.3.4. Drug Dissolution Performance

The drug dissolution test was implemented by the Chinese Pharmacopoeia 2015 edition pulp method. Drug-loaded fibers (10 mg) were placed into conical flasks containing 200 mL PBS buffer and 0.5% (*v*/*v*) Tween 80 (pH 6.7), and the SHA-AB shaking incubator provided a constant water bath environment of 37.5 °C and a constant oscillation rate of 50 rpm. The sample (4 mL) was collected within the established time interval and mark and then supplemented with a fresh equal amount of release medium (*n* = 3). The absorption peak intensity of the internal drug-containing solution was obtained using a UV–vis spectrophotometer (UV-2102PC, Unico Instrument Co., Ltd., Shanghai, China). Samples were carried out under the condition of removing the blank sample background (pure-release medium). For the Cur standard curve, 40 μg·mL^−1^ mother liquor was gradually diluted to obtain a concentration of 0.5 μg·mL^−1^, and the absorption peak of 0.5–40 μg·mL^−1^ was obtained at λ_max_ = 425 nm by UV–vis spectrophotometry (Figure S1 in the Supplementary Materials). For actual drug content, accurately weighed 10 mg drug-loading fibers were dissolved in the organic solvent and diluted to 200 mL by using a release medium. The actual total amount of drug was easy to obtain through UV–vis spectrophotometry and standard curve. All experiments were conducted thrice, and drug data were presented as mean ± standard deviation.

The first-order kinetic model (*A*) was to evaluate the linear relationship between drug release and relevant time. And the Higuchi model (*B*) was used to explore the drug dissolution and diffusion.

*A* = *a* (1 − *e*^−*b t*^) (1)

*B* = *c* + *d t*^1/2^ (2)

where *A* and *B* are the cumulative drug release at the time *t*, *a* and *c* indicate the initial drug amount, *b* and *d* are the constant in the first-order kinetic and Higuchi model, respectively. 

The well-known Rigter–Peppas equation was used to analyze drug dissolution kinetics:

*Q* = *M_t_*/*M*_∞_ = *kt^n^* (3)

The modified form based on Equation (3) was as follows:

*log Q* = *m* + *n log t* (4)

where *Q* is the relative release of Cur, *M_t_* is the relative release of Cur at time *t*, *M*_∞_ is the relative release of Cur at time ∞, both *k* and *n* are coefficients, *k* is a constant, *n* is the characteristic parameters of the dissolution mechanism, and *m* is the logarithms of *k*.

## 3. Results and Discussion

### 3.1. Coaxial Electrospinning

Traditional single and coaxial electrospinning can easily manufacture the working fluid from liquid to solid fiber under the effect of electrostatic fluid (Figure 1a exhibits). In this work, the homemade spinneret was a key component to fabricate double-layer or complex multilayer structures. The homemade spinneret (in the insert picture in Figure 1a), which consists of two metal capillaries (14 G and 17 G), was encapsulated, and fixed by epoxy resin.

For a successful electrospinning process, the electrostatic repulsion between the same charge forces the droplets (from the working fluids) to form a Taylor cone. Subsequently, the solvent in the working fluid was quickly evaporated through stretching and thinning processes (unstable whipping area). Finally, the formed fibers were collected at a certain distance (usually 10–20 cm) [53,54]. In Figure 1b,c, the powerful dyeing effect of Cur causes a yellow Taylor cone at the spinneret. Compared with the clear yellow Taylor cone (Figure 1b), a pale-yellow core–shell Taylor cone (Figure 1c) could be attributed to the white PHBV working fluid in the outer layer. In traditional single-fluid electrospinning (preparation of F1 fibers, Figure 1b), a gelatinous material appeared at the Taylor cone, which leads to the instability of the fiber fabrication process. The stable preparation process of F2 fibers (Figure 1c) was due to the appearance of the semiarch-shaped Taylor cone.

### 3.2. Morphological Characterization of the Fiber Surface

**Figure 2:**
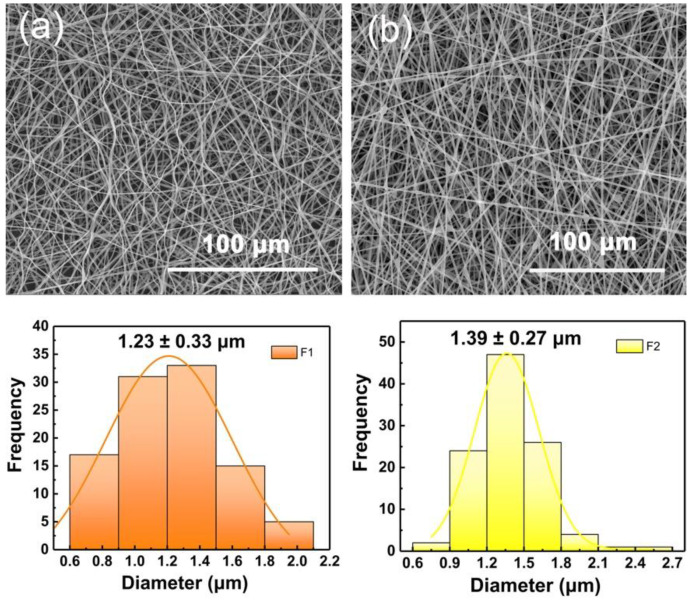
SEM images of (**a**) F1 and (**b**) F2 fibers. The average fiber diameter is distributed directly below the corresponding SEM image.

The SEM images of the two fabricated fibers in Figure 2a,b exhibited a linear cylindrical appearance. In Figure 2a, F1 fibers showed a curved and curled surface. This result could be predicted by combining with the digital picture of the electrospinning process in Figure 1b. In traditional single-fluid electrospinning, the gel substance appearing at the spinning nozzle caused interfacial surface tension and static electricity to fail to reach a dynamic equilibrium. Thus, the unexpected unstable electrospinning process caused fiber bending and curling. In Figure 2b, F2 fibers showed a straight fiber morphology with occasional beads, which could be attributed to the effect of PHBV working fluid in the outer layer. The electrospinning ability of pure PHBV solution to stretch into fibers is weak. Thus, the pure PHBV solution could not form fibers effectively (Figure S2 in the Supplementary Materials). However, in this work, the addition component of PHBV (as a shell layer) optimizes the electrospinning process (Figure 1c) and increased the diameter of fibers (average diameters of F1 and F2 are 1.23 ± 0.33 and 1.39 ± 0.27 μm, respectively). In Figure S4 in the Supplementary Materials, the broken F2 fiber showed a clear core-shell structure, and the extra PHBV out layer led to an increased diameter.

### 3.3. Phase and Compatibility Analyses

**Figure 3:**
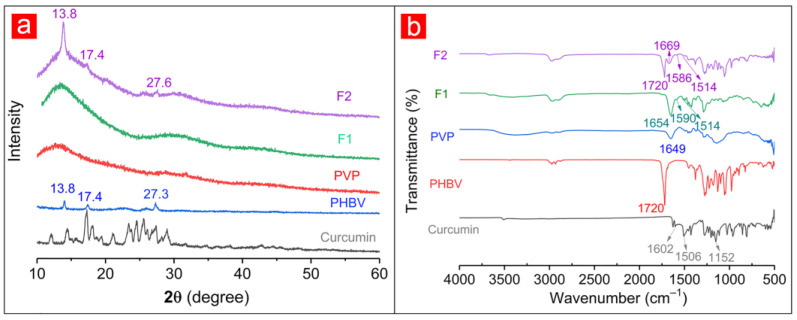
XRD patterns (**a**) and ATR-FTIR spectra (**b**) of raw materials (i.e., PVP, PHBV, and Cur) and fibers (i.e., F1 and F2).

The XRD patterns of raw materials (i.e., Cur, PHBV, and PVP) and fibers were shown in Figure 3a. A considerable number of Bragg diffraction peaks appearing in the XRD pattern of Cur suggested the crystalline properties of the Cur drug. Similarly, several diffraction peaks in the XRD pattern of PHBV could indicate the crystal structure of PHBV. Among them, the peaks at 13.8° and 17.4° indicated the α phase of PHBV and were attributed to the (020) and (110) crystal planes of PHB, respectively. Additionally, 2*θ* = 27.3° was attributed to the (121) crystal planes of PHB [55]. By contrast, the smooth XRD pattern suggested that PVP was amorphous. Although F1 fibers contained Cur, its XRD pattern had no sharp diffraction peaks like PVP. Similarly, apart from the three diffraction peaks of the known crystal PHBV in the F2 fibers’ XRD pattern, no other diffraction peak was related to Cur. During the electrospinning process, the crystalline drug was converted into an amorphous form. The drug-containing working fluid was stretched and solidified into fibers under the action of static electricity within several seconds at the spinning nozzle, and the drug was quickly fixed on the solid fiber during this period, thus losing its original crystal form [56].

The IR spectra of raw materials and drug-loaded fibers were obtained to explore the possible compatibility of components (Figure 3b). The characteristic peaks at 3510, 1602, 1506, and 1152 cm^−1^ of Cur demonstrate hydroxyl (-OH), carbonyl (-C=O), olefins (-C=C-), and ether (-C-O) groups, respectively [45,57,58]. The characteristic -C=O groups of PHBV and PVP were observed in 1720 and 1649 cm^−1^, respectively [59,60]. For F1 fibers, the characteristic peak at 1654 cm^−1^ represented the -C=O group of PVP, and those at 1590 and 1514 cm^−1^ corresponded to the -C=O and -C=C- groups of Cur. Similarly, the characteristic peaks of PHBV (1720 cm^−1^ for -C=O), PVP (1669 cm^−1^ for -C=O), and Cur (1586 cm^−1^ for -C=O, 1514 cm^−1^ for -C=C-) appeared in the F2 fibers’ IR spectra. The characteristic peaks of Cur showed a deviation on F1 and F2 curves, suggesting that the formation of hydrogen bonds between the -OH group of Cur molecule and -C=O group of PVP molecule. The generation of hydrogen bonds promoted compatibility between Cur and polymer matrix, thus improving the stability of fibers.

### 3.4. Performance of Functional Fibers

**Figure 4:**
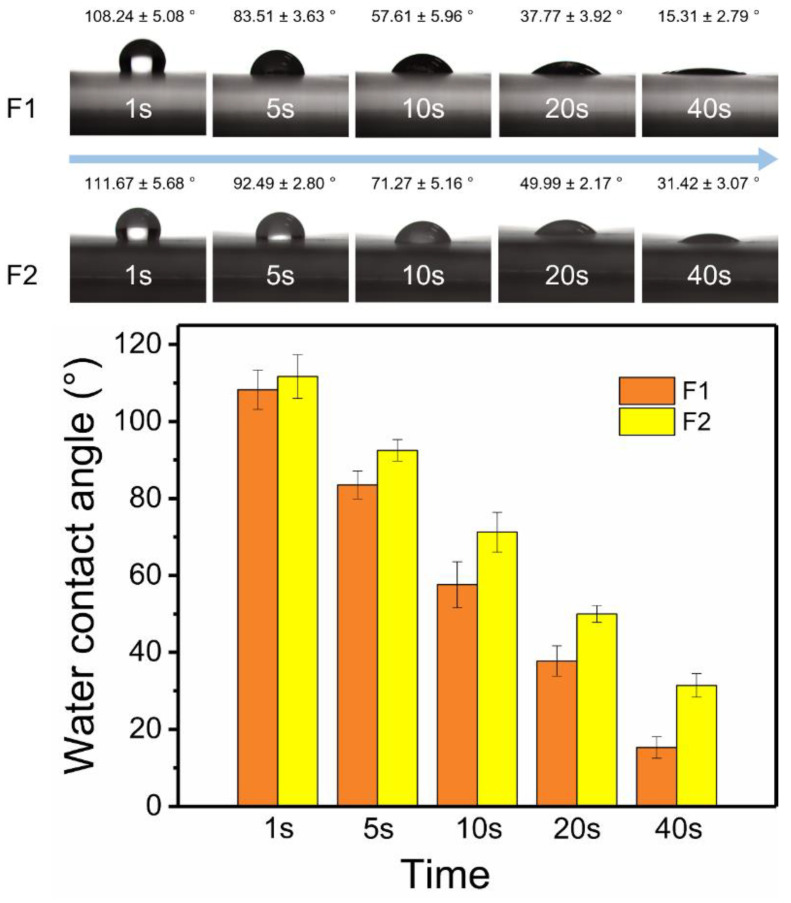
Water contact angles of F1 and F2 fibers in 40 s.

The surface hydrophilic properties of fibers were shown in Figure 4. In the first second, the water contact angle of F2 fibers (111.67° ± 5.68°) was slightly larger than that of F1 fibers (108.24° ± 5.08°). As time went by, the water contact angles of F1 and F2 fibers, which were 15.31° ± 2.79° and 31.42° ± 3.07°, respectively, gradually became smaller and stable at 40 s. The hydrophilic properties of two fibers were provided by PVP, and PHBV was responsible for the weaker hydrophilic properties of F2 fibers. The outer layer prevented the water molecules from entering the inner PVP layer, thereby delaying the intrusion of water molecules. More details could be observed on the digital pictures of the fibers after the water contact angle test. As shown in Figure S3 in the Supplementary Materials, the aluminum foil and dissolved Cur were exposed due to the water droplets dissolve the PVP matrix of F1 fibers. By contrast, water had left a mark in the form of a core–sheath on the fiber mat (dark color in the inner layer and light color in the outer layer) in F2 fibers. This intuitive evidence suggested that the outer PHBV layer could inhibit water molecules from entering fibers.

**Figure 5:**
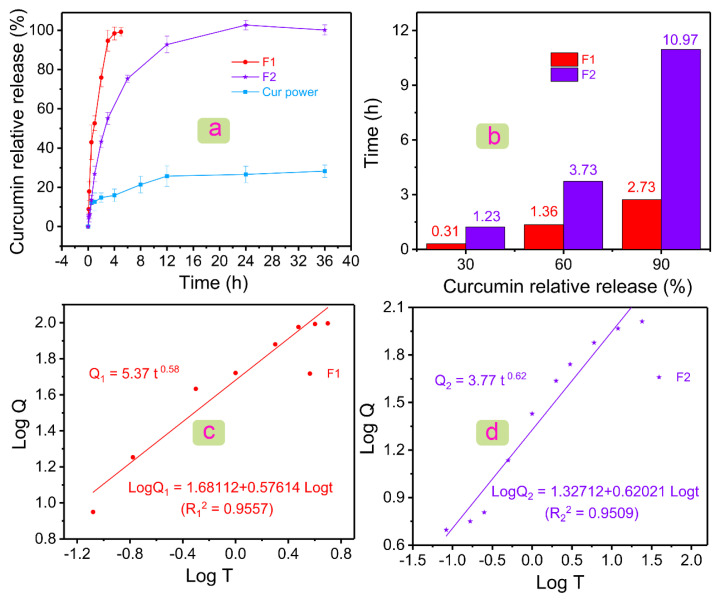
Drug dissolution results and analysis. (**a**) Relative release profiles of Cur powder, F1 fibers, and F2 fibers in 36 h. (**b**) Histogram of the relationship between Cur relative release and time in F1 and F2 fibers. Linear relationship between *Log T* and *Log Q* of (**c**) F1 and (**d**) F2 fibers, where *Q* is the relative release amount of Cur.

Figure 5a showed the drug dissolution profiles of Cur powder, F1 fibers, and F2 fibers. For poorly water-soluble Cur, it showed a burst release of 12.05 ± 3.66% in the first half hour, and only 28.26 ± 3.16% of poorly water-soluble Cur could be dissolved in 12 h. The lower solubility could be improved by the prepared two fibers. The F1 fiber could release 99.19 ± 2.24% Cur in only 4 h. PVP, a commonly used drug carrier, played an important role in the rapid release of poorly water-soluble drugs [61,62,63]. However, the rapid deposition of local Cur (hard to absorb) affects the body. Thus, the sustained and stable release of Cur is needed. The coaxial electrospun fibers designed in this study could delay the release rate of Cur under the premise of ensuring the efficient release of Cur. F2 fibers released 100.14 ± 2.58% Cur in 36 h and had no initial burst stage. Drug molecules were stored in the core of fibers, and drug release was delayed due to the obstruction of the outer polymeric layer. Therefore, there was no initial burst release in F2 fibers. The combination of core–shell structure and raw polymeric materials (i.e., PHBV and PVP) not only improved the solubility of Cur, but also made it have a sustained-release drug profile of more than 24 h.

Figure 5b showed the relationship between the time required and the amount of Cur released at a certain stage was given. F2 fibers needed 1.23 h to release 30% Cur, whereas F1 fibers only needed 0.31 h. In the middle of the release of Cur (60%), F1 and F2 fibers needed 1.36 and 3.73 h, respectively. Subsequently, F1 fibers could release 90% Cur in 2.73 h, and F2 fibers needed 10.97 h. After the release of 90% Cur, F2 fibers needed a longer time (24 h) to release Cur completely compared with F1 fibers (4 h). Differentiated drug data showed the excellent performance of F2 fiber in the sustained release of the poorly water-soluble drug Cur.

Drug data were further processed by several mathematical models (i.e., first-order kinetic model, Higuchi model, and Rigter–Peppas model), such as Table 1. Drugs in the two fibers could be released at a constant percentage rate, because of the higher correlation coefficient *R*^2^ in the first-order kinetic model. The Higuchi model was used to describe the dissolution and diffusion behavior of drugs from the drug delivery systems. The higher *R*^2^ indicated the two drug-loaded fibers had a diffusion release mechanism. The Rigter–Peppas equation was used to analyze the drug release kinetics deeply (Figure 5c,d) [64]. The equations for F1 and F2 fibers were *Q*_1_ = 5.37 *t*^0.58^ (*R*_1_^2^ = 0.9557) and *Q*_2_ = 3.77 *t*^0.62^ (*R*_2_^2^ = 0.9509), respectively. *n* in *Q*_1_ and *Q*_2_ were 0.58 and 0.62, which were both in the range of 0.45–0.9. In general, it indicated that F1 and F2 fibers had both Fickian diffusion and skeleton erosion mechanisms. In fact, the drug was all stored in the PVP matrix inside the F1 and F2 fibers, and PVP was a water-soluble polymer; thus, F1 and F2 fibers should have a skeleton erosion mechanism. The difference between Peppas equations of F1 and F2 was not obvious, except for the slightly increased of *n* value in F2 fibers. This suggested that the addition of the component PHBV layer had no adverse effect on the drug release kinetics in the fibers.

### 3.5. Mechanism of Drug Release in Fibers

**Figure 6:**
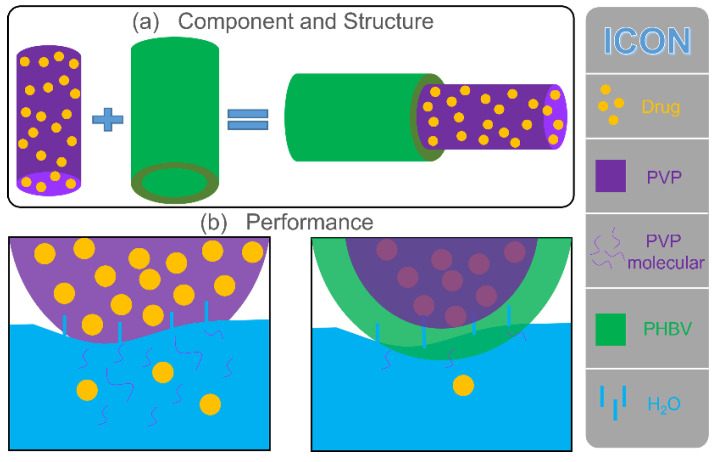
Mechanism of drug release in F1 and F2 fibers. (**a**) Component and structure of F1 and F2 fibers. (**b**) Schematic of the drug release of F1 and F2 fibers in water.

The drug release behavior was remarkably affected by the physicochemical properties of polymers. In this study, the PVP polymeric matrix, a common drug-loading carrier, had natural advantages for encapsulating some poorly water-soluble drugs. The rapid water solubility of PVP provided an effective solution for the release of poorly water-soluble drugs. The fabricated monolithic F1 fiber was composed of PVP, and the drug was evenly distributed on its surface and inside. As shown in Figure 6, water molecules penetrated F1 fibers from all directions to dissolve PVP quickly, and the drug active molecules diffused into the release medium due to the disappearance of the polymeric matrix. As an excellent drug carrier, PVP could load most of the drugs in the pharmacy field for rapid release. The prepared F2 fibers added a PHBV layer based on F1 fibers during the structure design. Therefore, the penetration of water molecules and the diffusion of drugs were more complicated than direct contact (F1 fibers). First, water molecules slowly penetrate the PHBV layer through diffusion and reached the inner PVP layer. Water molecules carried a small number of PVP molecules, and drug molecules returned to the release medium along the original path of diffusion. Finally, as the PHBV layer was completely penetrated by water molecules, a considerable number of PVP molecules and drug molecules slowly passed through the PHBV layer to the release medium along with the diffusion of water molecules. Compared with F1 fibers, core–shell F2 fibers remarkably increased the diffusion distance of the drugs from the storage space to the medium.

The design of the core–sheath structure and raw material PHBV changed the hydrophilicity of fibers (large water contact angle showed in Figure 4), which inhibited the diffusion of water to a certain extent and delayed the release of drugs (Figure 5), thus affecting the diffusion mechanism of drug molecules. The good improvement in material performance due to structure and raw materials had released a positive signal in the drug release system, providing a reference for future customized drug fibers.

## 4. Conclusions

In this study, a combination strategy of hydrophilic PVP and hydrophobic PHBV was used to prepare coaxial fibers, which have the properties of optimizing the release of poorly water-soluble drug. The PHBV with poor fiber forming performance, as a sheath layer, was used to prepare core–shell fibers by coaxial electrospinning under the drive of PVP with Cur. Core–shell F2 fibers showed a straight cylindrical appearance with few spindles under SEM, whereas monolithic F1 fibers showed a curved linear morphology. The results of XRD and Fourier transformed infrared spectroscopy suggested that the model drug Cur, which has an amorphous form, was uniformly distributed in fibers and had good compatibility with the polymeric matrix. The decrease in the hydrophilicity of F2 fibers (from 31.42° ± 3.07° to 15.31° ± 2.79°) is due to the addition of the hydrophobic PHBV component and the design of the core–shell structure. During in vivo drug release, F1 fibers only needed 4 h to release Cur completely due to the water-soluble PVP, and core–shell F2 fibers could prolong the Cur release time to 24 h. This long-term dosing method remarkably improves the therapeutic utilization efficiency of poorly water-soluble Cur. This drug loading method combined with the properties of PVP, and the design of the fiber structure provide a new solution for drug delivery systems and could be useful in exploring novel and effective biomedical materials.

## Figures and Tables

**Table 1:** Mathematical model analysis of drug release data for prepared fibers.

Samples	First-Order Kinetic Model (*R*^2^)	Higuchi Model (*R*^2^)	Rigter–Peppas Model (*R*^2^)
F1	*A*_1_ = 100.08(1 − e^−0.85 *t*^) (*R*_1_^2^ = 0.9796)	*B*_1_ = 48.35 *t*^1/2^ + 2.18 (*R*_1_^2^ = 0.9561)	*Q*_1_ = 5.37 *t*^0.58^ (*R*_1_^2^ = 0.9557)
F2	*A*_2_ = 99.51(1 − e^−0.27 *t*^) (*R*_2_^2^ = 0.9955)	*B*_2_ = 19.07 *t*^1/2^ +8.25 (*R*_2_^2^ = 0.8640)	*Q*_2_ = 3.77 *t*^0.62^ (*R*_2_^2^ = 0.9509)

## Data Availability

The data supporting the findings of this manuscript are available from the corresponding authors upon reasonable request.

## Supplementary Materials

The following supporting information can be downloaded at: https://www.mdpi.com/article/10.3390/polym14030469/s1, Figure S1: The drug standard profile of curcumin after linear fitting.; Figure S2: SEM images of 9% and 15% PHBV products; Figure S3: Digital picture of the F1 (a) and F2 (b) fibers surface after water contact angle test; Figure S4: SEM image of the broken F2 fiber.
