# Supplementary material for: Electrospun Coaxial Fibers to Optimize the Release of Poorly Water-Soluble Drug
Source: Polymers (Basel). 2022 Jan 24;14(3):469. doi: 10.3390/polym14030469 (PMC8839822; doi:10.3390/polym14030469)
Supplement: Supplementary file 1 [file polymers-14-00469-s001.zip › polymers-1564015-supplementary.pdf]

Supplementary Materials

Electrospun Coaxial Fibers to Optimize the Release of Poorly Water-Soluble Drug

*Yubo Liu [1], Xiaohong Chen [1,2], Yuyang Liu [1], Yuhang Gao [1], Ping Liu [1, 2, *]*

[1] School of Materials and Chemistry, University of Shanghai for Science & Technology, Shanghai 200093, China

[2] Shanghai Engineering Technology Research Center for High-Performance Medical Device Materials, Shanghai 200093, China

* Corresponding author：

Prof. Ping Liu

School of Materials and Chemistry

University of Shanghai for Science and Technology

Address: 516 Jungong Road, Yangpu District, Shanghai 200093, China

Email: liuping@usst.edu.cn

Tel: +86-021-55271656

1. The flow rate conversion process is as following:

In the Yunfan spinning machine, the flow rate of working fluids is indirectly controlled by the forward speed of the pump. To avoid unnecessary misunderstandings, the following equations were used to convert it into a conventional flow unit.

$V = A_1 \times T \times \pi \times (D/2)^2 \times 10^{-3}$. (mm/s) × (s) × (mm^2) × 10^{-3}= (cm^3) = (ml). (1)

$A_2 = V / T \times 3600$. (ml) / (s) × 3600 = (ml/h). (2)

Where V is the volume pushed by the syringe pump at the time T, A_1 is the propulsion rate of the syringe pump in the system, D is syringe barrel diameter, and A_2 is the flow rate.

The diameter D of the 10 ml syringe barrel is 15 mm, and the propulsion rate of the syringe pump is 0.0014 mm/s. Therefore, with the help of formula 1 and 2, we can get that:

$V = 0.0014 \times T \times \pi \times (15/2)^2 \times 10^{-3} \approx 2.474 \times 10^{-4}\, T$ (ml)

$A_2 = V / T \times 3600 \approx 0.9$ (ml/h)

Similarly, when the advancing speed of the syringe pump is 0.0022mm/s, the converted pushing speed is 1.4ml/h.

2. The drug standard curve of curcumin

Curcumin powders were dissolved in a 500 ml PBS buffer containing 0.5% v/v Tween 80 (pH 6.7) to prepare a 40 μg/ml of curcumin mother liquor. Subsequently, the mother liquor is gradually diluted into a drug solution of the required concentration, and the absorption intensity of curcumin solutions at λ=425 nm was obtained by using a UV-vis spectrophotometer. Finally, the standard curve is calculated by linear fitting.

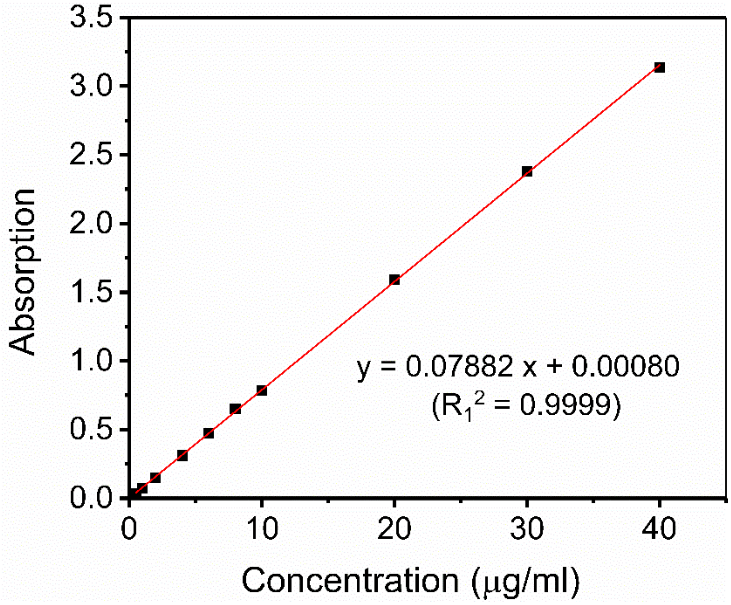

Figure S1. The drug standard profile of curcumin after linear fitting.

3. The surface morphology of PHBV

The PHBV products were fabricated by traditional single-fluid spinning, the SEM images showed no significant difference between the concentration of 9% and 15%, which both exhibited a coexistence of discontinuous fiber with beads.

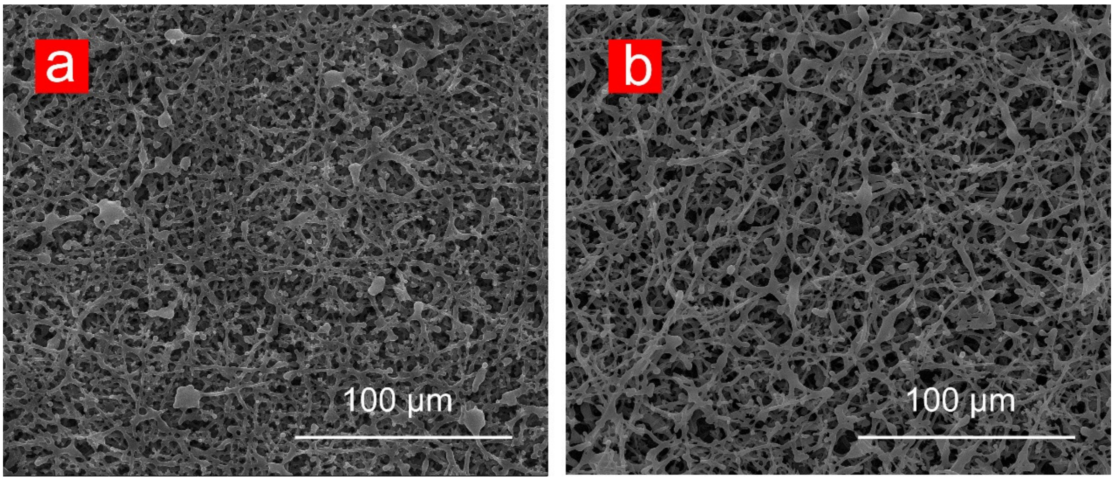

Figure S2. SEM images of 9% and 15% PHBV products.

4. The experiment of water contact angle

The surface picture of the two fibers after six experiments was exhibited in Fig. S3, which has completely different shapes. F1 fibers were completely dissolved near the water droplets, which the aluminum foil under the fiber and curcumin loaded in the fiber were completely exposed. In contrast, F2 fibers showed a tendency to spread from the inside out, which gives a hint of the formation of the core–shell structure.

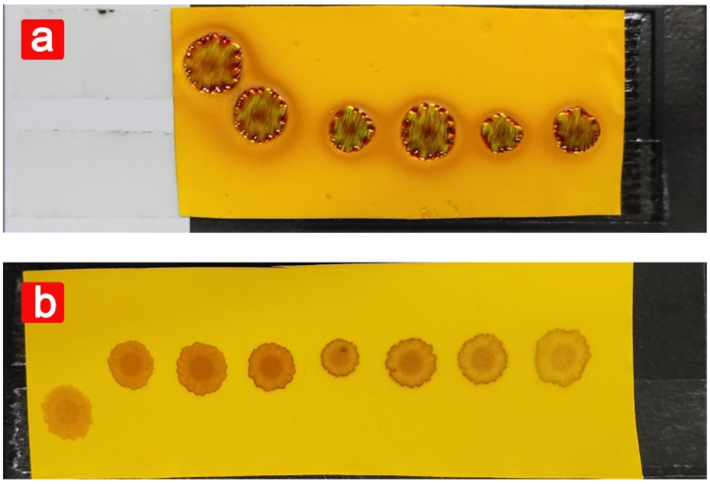

Figure S3. Digital picture of the F1 (a) and F2 (b) fibers surface after water contact angle test.

5. Broken fiber

To verify the core-shell structure, F2 fibers were placed in liquid nitrogen for several minutes and broken with tweezers. F2 fibers clearly exhibited a core-shell structure in Figure S4. The outer layer was broken, while the inner layer was intact.

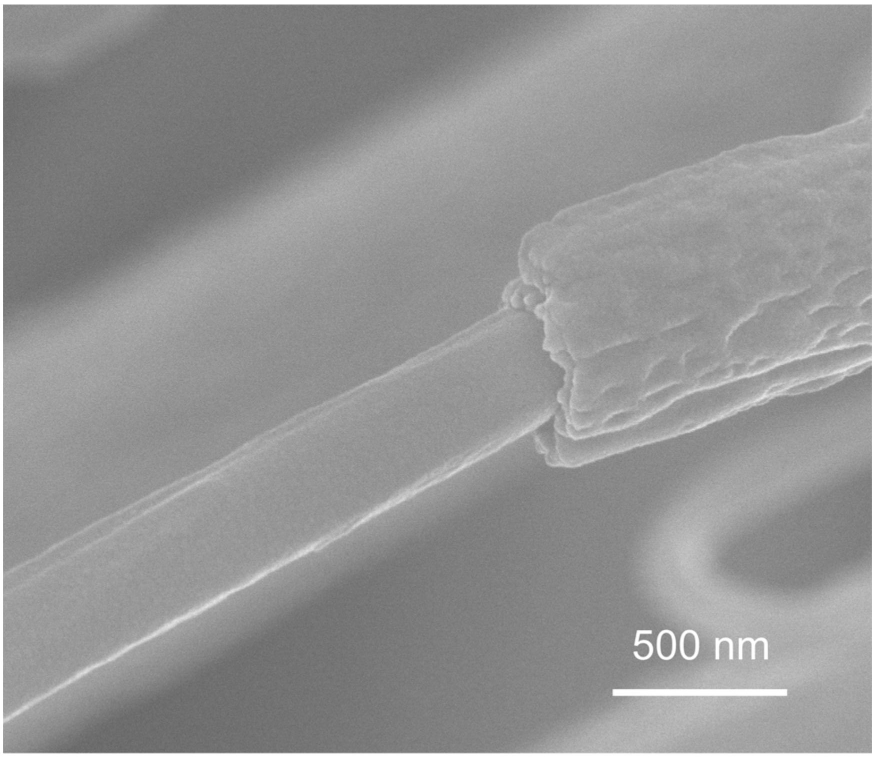

Figure S4. SEM image of the broken F2 fiber.
